# The Pioglitazone Trek via Human PPAR Gamma: From Discovery to a Medicine at the FDA and Beyond

**DOI:** 10.3389/fphar.2018.01093

**Published:** 2018-10-04

**Authors:** Pallavi R. Devchand, Tianyun Liu, Russ B. Altman, Garret A. FitzGerald, Eric E. Schadt

**Affiliations:** ^1^Department of Physiology and Pharmacology, University of Calgary, Calgary, AB, Canada; ^2^Department of Genetics, Stanford University, Stanford, CA, United States; ^3^Department of Bioengineering, Stanford University, Stanford, CA, United States; ^4^Department of Systems Pharmacology and Translational Therapeutics, Perelman School of Medicine, University of Pennsylvania, Philadelphia, PA, United States; ^5^SEMA4, a Mount Sinai Venture, Stamford, CT, United States; ^6^Icahn Institute of Genomics and Multiscale Biology, Icahn School of Medicine at Mount Sinai, New York, NY, United States

**Keywords:** pioglitazone, PPAR gamma, protein–drug interactions, nuclear receptor, glitazone medications

## Abstract

For almost two decades, pioglitazone has been prescribed primarily to prevent and treat insulin resistance in some type 2 diabetic patients. In this review, we trace the path to discovery of pioglitazone as a thiazolidinedione compound, the glitazone tracks through the regulatory agencies, the trek to molecular agonism in the nucleus and the binding of pioglitazone to the nuclear receptor PPAR gamma. Given the rise in consumption of pioglitazone in T2D patients worldwide and the increased number of clinical trials currently testing alternate medical uses for this drug, there is also merit to some reflection on the reported adverse effects. Going forward, it is imperative to continue investigations into the mechanisms of actions of pioglitazone, the potential of glitazone drugs to contribute to unmet needs in complex diseases associated with the dynamics of adaptive homeostasis, and also the routes to minimizing adverse effects in every-day patients throughout the world.

## Introduction

“I have discovered the great secret that after climbing a great hill, one only finds that there are many more hills to climb.”

- Nelson Mandela *in* The Long Walk to Freedom, 1994.

Type 2 diabetes (T2D) is a chronic disease characterized by insulin resistance. If left unchecked over time, T2D can progress to damage the kidneys, eyes, nerves, heart, and blood vessels. The consequent downstream results include increased risk of kidney failure, diabetic retinopathy, heart attacks, and strokes. Interestingly, the insulin resistance of this disease is itself often consequential of obesity and physical inactivity. Indeed lifestyle measures, like caloric restriction and aerobic exercise, can be effective in preventing or delaying the onset of T2D. Nevertheless the reality of the World Health Organization demographics is sobering: over 90% of diabetics have T2D; a global obesity epidemic has ensured that T2D it is no longer solely an adult onset disease; and T2D will likely be the seventh leading cause of death in 2030^1^. In America alone, an estimated ∼84.1 million adults are pre-diabetic^2^. The good news is that non-insulin-dependent diabetes mellitus (NIDDMs) can be treated and managed in several ways throughout life. Pioglitazone offers one such avenue that, like any medication, comes with risks and benefits.

Pioglitazone is also being evaluated globally, in over 50 clinical trials, for its potential use in unmet needs of several complex multifactorial diseases^3^. To gain some perspective on the roads ahead for pioglitazone, we analyze its history from discovery to clinic. The ever-present challenge remains to harness the potential of pioglitazone in modulating molecular mechanisms associated with adaptive homeostasis while also balancing the risks and benefits of this medicine in real world patients with unmet clinical needs.

### Glitazones as a New Class of Drugs

**FIGURE 1:**
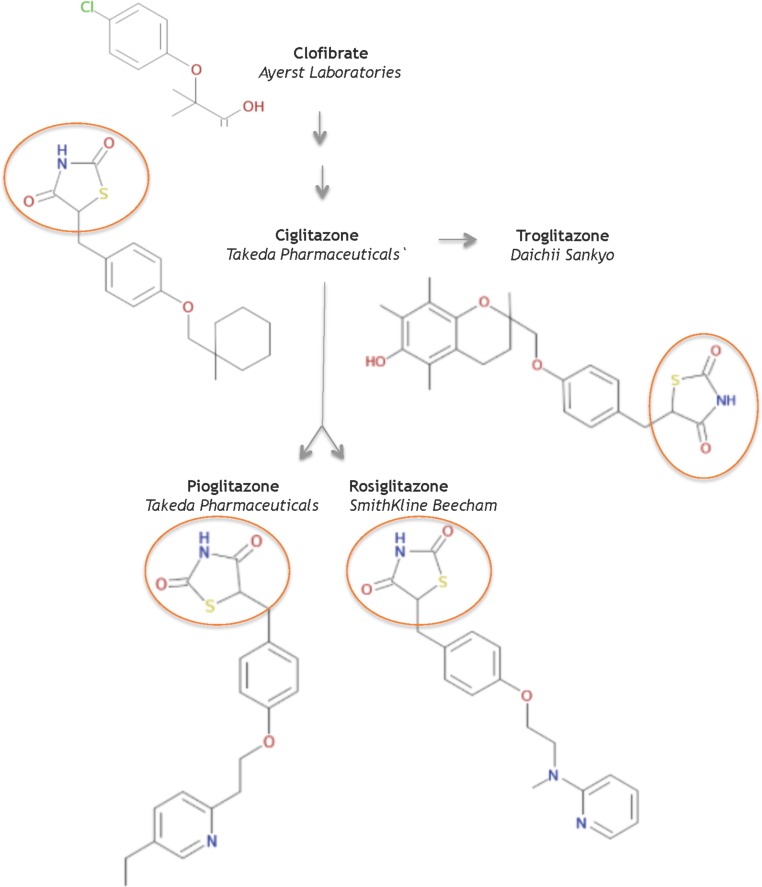
Structures of key compounds in pioglitazone discovery (Gaulton et al., 2012; Bento et al., 2014).

In 1982, scientists at Takeda Pharmaceuticals in Japan reported their continued efforts on a derivative compound library of the clofibrate class as novel lipid-lowering agents (Sohda et al., 1982b). They discovered that the acidic thiazolidine-2,4-dione (TZD) moiety can improve both the hypoglycemic and the hypolipidemic activity in yellow KK mice, a genetically obese mouse model for NIDDMs. In their second report, Sohda et al. (1982a) sought to improve TZD activity and discovered ADD-3878 (ciglitazone) as an anti-diabetic drug that had the unique ability to reduce blood glucose levels without increasing insulin secretion in the KKA^y^ mouse model. Further studies showed that ciglitazone also functioned to reduce peripheral insulin resistance in other rodent models of obesity and/or diabetes (Fujita et al., 1983). Thus the universal search was triggered for improved, more potent glitazones to medicate patients with NIDDMs. Ultimately, three investigational TZD drugs gained regulatory approval as medicines: troglitazone, rosiglitazone, and pioglitazone (**Figure 1**).

At Sankyo Co., in Japan, the drug CS-045 (troglitazone) stemmed from the hypothesis that the inhibition of lipid peroxidation would improve hypolipidemic and/or hypoglycemic drugs, since lipid peroxidation was partly responsible for the angiopathic diabetic complications (Yoshioka et al., 1989). Several series of hindered phenols were systematically investigated for inhibiting lipid peroxidation activity in rat liver microsomes, for lowering serum lipid peroxides in the ALLOXAN mouse model, as well as for hypolipidemic and hypoglycemic activity in various rodent models. These functional studies seeded troglitazone, a compound that combined ciglitazone with a Vitamin E component, as a candidate to pursue in clinical studies – in part, because troglitazone did not increase liver weight.

In 1994, scientists at SmithKline Beecham in the United Kingdom reported a series of urea analogs of ciglitazone that were designed to lower lipophilicity and increase potency (Cantello et al., 1994). Using the genetically obese mouse C57 Bl/6 *ob/ob* as a model for NIDDMs, these structure-activity relationship studies assayed for antihyperglycemic potency and for impact on blood hemoglobin profiles. This research discovered the aminopyridine BRL 49,653 (rosiglitazone). Follow-up studies in rodents indicated select differences in biological profile compared to the other glitazones, and defined rosiglitazone as a candidate for further study and clinical development (e.g., Oakes et al., 1994).

AD-4833 (Pioglitazone) was discovered at Takeda, using didactic studies on the cyclohexylmethyl group of their prototypical compound ciglitazone, with the primary aim of increasing potency (Sohda et al., 1990). The toxicological profile of pioglitazone proved superior to other compounds in this series of pyridine analogs (Meguro and Fujita, 1987). Further pharmacological studies in several rodent models and in aged, obese beagle dogs indicated that pioglitazone ameliorated insulin resistance phenotypes of abnormal glucose and lipid metabolism by enhancing insulin action on peripheral tissues (Ikeda et al., 1990). Based on its pharmacology and toxicology, pioglitazone was selected for clinical development to treat NIDDMs.

**FIGURE 2:**
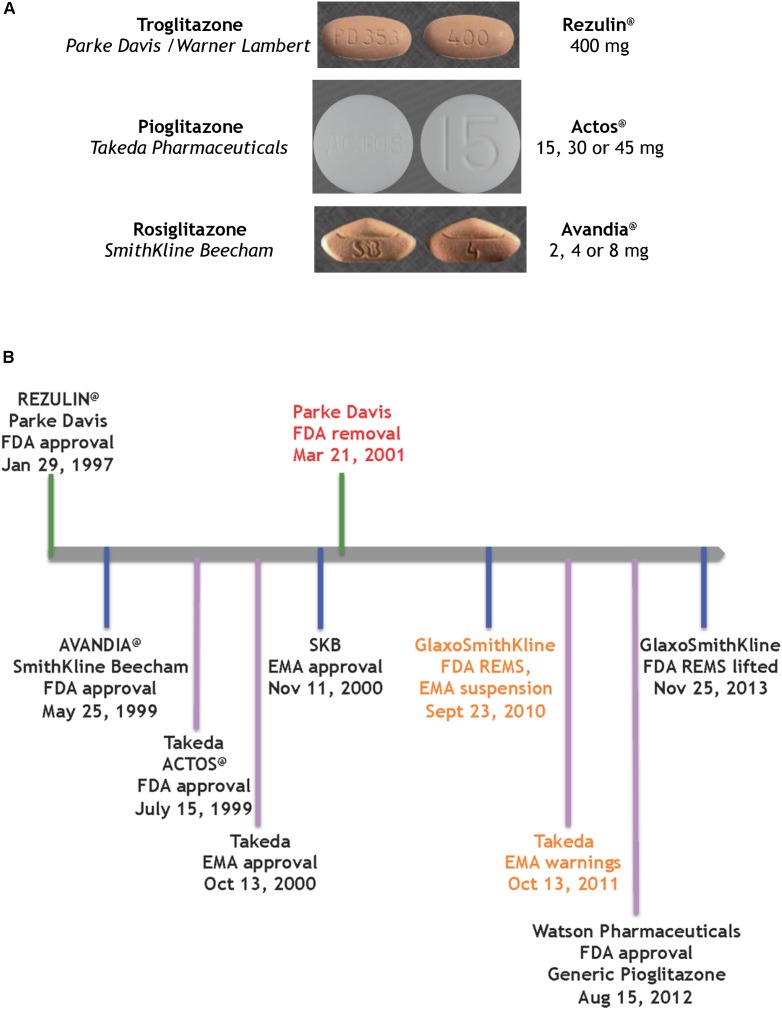
Glitazones as medicines **(A)** the glitazone pills (Wishart et al., 2018), **(B)** timeline of key regulatory events for the glitazones.

In summary, the systematic evolution of ciglitazone from clofibrate led to the discovery of TZDs as a novel class of antidiabetics that improved hypoglycemia and hypolipidemia without impacting the secretion of insulin from the pancreas. Pioglitazone was derived from targeted improvements of the insulin-sensitizing potency of ciglitazone, with added benefit on hemoglobin profiles. It is one of three glitazones that became medicines (**Figure 2A**).

### Life on the FDA Fast Track

In the early 1990s, the world was challenged with a burgeoning of unmet medical needs and life-threatening diseases, most notably AIDS. In response to these crises, the U.S. Food and Drug Administration (FDA) instituted the Accelerated Drug Approval Program that effectively facilitated expedited approvals for life-saving drugs using surrogate end-points as a valid measure of clinical benefit. For example, a primary endpoint to measure for drug effectiveness in T2D was the change in blood levels of HbA_1c_ from baseline. As a minor component of hemoglobin bound to glucose, HbA_1c_ levels do not display the daily variations of glucose in the blood, and have the advantage of being insensitive to short term variations in blood glucose while reflecting glucose levels of the past 6–8 weeks. Importantly, this practical route of the FDA expedited drug approval is dynamically monitored in a Phase IV clinical trial – essentially a post-marketing monitoring of drug safety in the long-term.

The first drug candidate approved on this FDA track was a glitazone for the treatment of T2D by reducing insulin resistance (**Figure 2B**). Troglitazone was licensed, produced and marketed by Parke Davis in America. REZULIN^@^ was submitted for review to the FDA on July 31st, 1996; approved on January 29th, 1997 and marketed in March of 1997. Plagued by warnings of idiosyncratic liver failure, on October 27th, 1997 the FDA mandated a warning on the drug label requiring monthly monitoring of liver enzyme levels. In June 1998 the Diabetes Prevention Program at the NIDDK halted its troglitazone investigational clinical study. Ultimately, with the advent of newer glitazones, REZULIN^@^ was voluntarily withdrawn from the market on March 21th, 2000. Of note, Glaxo Wellcome had licensed troglitazone for Europe and Britain, and introduced Ramozin^@^ in October 1997; followed by a rapid and voluntarily withdrawal on December 1st, 1997.

The melodramatic rise and fall of REZULIN^@^ paved a fast track for introduction of pioglitazone and rosiglitazone as medications for the treatment of T2D globally. SmithKline Beecham received FDA approval for AVANDIA^@^ on May 25th, 1999 but the drug faced a rocky ride on the Phase IV stage (ClinicalTrials.gov Identifier: NCT00000620). A claim that the drug elevated risks of heart attacks (Nissen and Wolski, 2007) ultimately provoked a FDA decision to restrict access to AVANDIA^@^. Further dramatic political intervention by members on the United States Senate Committee on Finance effectively terminated the ongoing TIDE clinical trial – a randomized controlled trial which included a head-to-head comparison of pioglitazone and rosiglitazone on the composite endpoint of myocardial infarction, stroke or cardiovascular death in diabetic subjects who were at risk of cardiovascular disease (ClinicalTrials.gov Identifier: NCT00879970). Three years later, an objective re-examination of the clinical data from the Action to Control Cardiovascular Risk in Diabetes (ACCORD) trial failed to support the notion that rosiglitazone elevated cardiovascular risk and thus resulted in lifting of the FDA REMS on AVANDIA^@^ (Woodcock et al., 2010) – perhaps a pyrrhic victory given that the earlier controversy had effectively undermined its future in the clinic.

ACTOS^@^ (*Takeda Pharmaceuticals*) was approved by the FDA on 15th July, 1999 and by the European Medicines Agency (EMA) on 13th October, 2000. The PROspective PioglitAzone Clinical Trial In MacroVascular Events (PROACTIVE) indicated reduced major adverse cardiovascular events associated with this glitazone (International Standard Randomized Controlled Trial, number ISRCTN NCT00174993). However, based on a report by Azoulay et al. (2012), the EMA placed warnings and contraindications of increased risk of bladder cancer. The drug remained on the market in Europe, to be used as an option when other therapies were ineffective. Of note, on August 15th, 2012 the FDA approved a generic version of pioglitazone (*Watson Pharmaceuticals*).

In summary, ∼20 years ago pioglitazone (ACTOS^@^, *Takeda Pharmaceuticals*) was approved by the FDA on an expedited track that was laid by REZULIN^@^. Currently pioglitazone is prescribed as a mono-therapy or oral combination for T2D, usually to patients who are overweight and when metformin is not a suitable option.

### Agonists as Medicines

**FIGURE 3:**
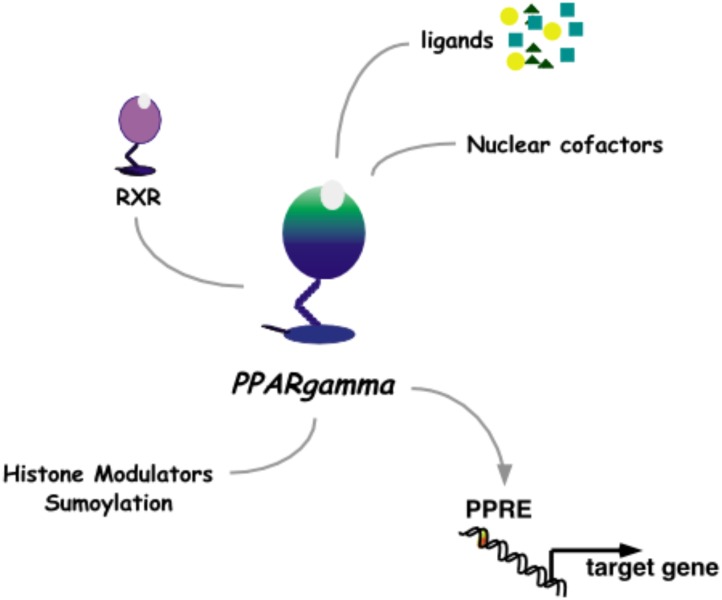
Nuclear receptor PPARγ. As a heterodimer with RXRγ, PPARγ mediates transcriptional activation through a PPRE DNA response element in promoters of target genes. This activity is modulated by binding to ligands and nuclear cofactors, as well as fine-tune regulation by sumoylation and histone modifiers.

The pharmacological approach to discovery of pioglitazone was aimed at uncoupling the lowering of blood glucose from the unwanted ensuing hypoglycemia that was characteristic of T2D medications at the time. While insulin and the sulphonylureas modulate signaling from the pancreas to peripheral tissues and the α-glucosidase inhibitors effectively decreased systemic glucose traffic, pioglitazone paved an alternate path by increasing insulin responsiveness in peripheral tissues. Once this novel pharmacology of pioglitazone was detailed as a clinical benefit, the challenge was to understand the molecular mechanisms of action. The first molecular target identified for pioglitazone was PPARγ (Peroxisome Proliferator-Activated Receptor gamma, **Figure 3**).

Some hypolipidemic drugs, like clofibrate, evoke a predictable and pleiotropic response in rodent hepatocytes that includes the ability to rapidly induce expression of key peroxisomal enzymes with concomitant increase in the number of peroxisome organelles. Lalwani et al. (1987) reported the purification of PPbP, a ∼70 kD protein from rat liver cytosol that was able to bind to hypolipidemic drugs of the clofibrate class. Subsequently, in a directed search for a nuclear hormone receptor, Issemann and Green (1990) cloned Peroxisome Proliferator-Activated Receptor (PPAR) from a rodent liver cDNA library and demonstrated the PPAR ligand-binding domain (LBD) as a potential transducer of non-genotoxic carcinogens like clofibrate. Dreyer et al. (1992) cloned three PPAR isotypes (α, β, and γ) from *Xenopus laevis* and delineated the PPRE, a DNA response element that mediated rapid induction of transcription by PPARs.

A pivotal body of work by Kletzien et al. (1992a) characterized pioglitazone-responsiveness in the process of insulin-dependent differentiation of mouse 3T3-L1 pre-adipocyte cells to adipocytes; demonstrated that pioglitazone-induced transcription of the adipocyte fatty acid binding protein via a DNA promoter segment was responsible for adipocyte-specific expression (Kletzien et al., 1992b); and delineated the DNA element ARE6 as a protein-binding sequence that was uniquely able to transmit pioglitazone responsiveness in a tissue-specific and differentiation-dependent manner (Harris and Kletzien, 1994). The 32bp ARE6 (^5′^TCGGCGCCATTTCACCCAGAGAGAAGGGATTG^3′^) was previously characterized by Graves et al. (1991) as a duplex DNA sequence that specifically bound to the protein factor ARF6, a protein detected in nuclear extracts of adipocytes and potentially a master regulator of adipogenesis. In 1994, the Spiegelman team purified the mouse ARF6 factor and, based on homology to known proteins, identified it as a heterodimer of the nuclear hormone receptors PPARγ/RXRα (Tontonoz et al., 1994a). They proposed that the isoform PPARγ2, which has an additional 30aa at the amino-terminal of the DNA-binding domain of PPARγ1, was primarily responsible for adipocyte-specific PPARγ activity in mouse (Tontonoz et al., 1994b). Interestingly, in transient transfection experiments performed in CV-1 monkey kidney cells, both murine PPARγ1 and PPARγ2 responded to pioglitazone with the same half-maximal effective concentration of activation (Lehmann et al., 1995). Furthermore, radiolabelled competition binding experiments using bacterially expressed fusion protein verified that pioglitazone was indeed a ligand for the murine PPARγ-LBD (Lehmann et al., 1995).

While the anti-diabetic activity of pioglitazone on PPARγ was demonstrated as agonistic, an anti-inflammatory activity via pioglitazone-PPARγ has been characterized as an indirect mechanism of squelching nuclear co-factors for nuclear factor KB (Ricote et al., 1998). Pioglitazone has also been shown to act as a weak agonist of the human PPARα isotype during transient transfection experiments in COS-1 immortalized monkey kidney cells, and as a ligand of the human PPARα-LBD that can induce secretion of Apolipoprotein A1 from HepG2 cells (Sakamoto et al., 2000). Interestingly, in a small cohort of T2D patients, pioglitazone dampened the expression of sVCAM-1 (soluble vascular cell adhesion molecule 1), a peptide associated with early atherogenic events (Orasanu et al., 2008). Trans-repression of sVCAM-1 expression was delineated using the PPARα-deficient mouse (Orasanu et al., 2008), a seminal animal model on the involvement of PPARs in xenobiotic responsiveness (e.g., Lee et al., 1995) and on the progression of a successful inflammatory defense (e.g., Devchand et al., 1996). Thus, the broader impact of pioglitazone-binding proteins on treatment of diabetes remains an active area of investigation (Colca et al., 2004; Devchand, 2008; Tamir et al., 2015).

In summary, understanding the molecular mechanisms of action of pioglitazone is an on-going challenge. As we learn more about the molecular dynamics of PPAR activity, it is clear that the pioglitazone responses in man are pleiotropic, nuanced by a web of molecular regulators and determined by both environment and individual health state. To date, the most studied molecular, aspect of pioglitazone is its function as an agonist and ligand of the nuclear receptor PPARγ.

### Pioglitazone in PPARγ

**FIGURE 4:**
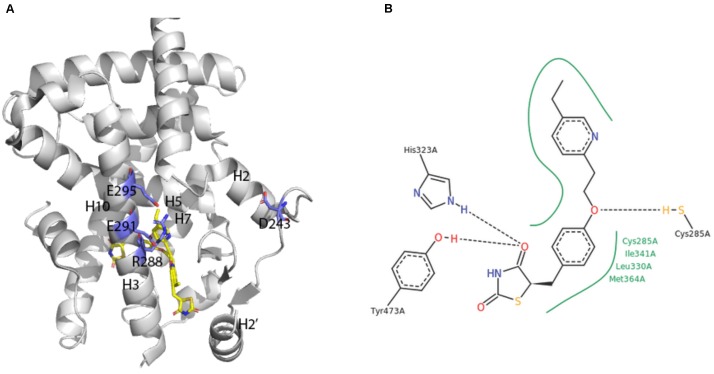
X-ray crystal structure of PPARγ ligand binding domain **(A)** The apo-human PPARγ recombinant protein structure (1PRG deposited by Nolte et al., 1998) was obtained from the RCSB protein database (Berman et al., 2000). Key placements of helix 3 (H3), as well as key hydrophilic and hydrophobic residues at the surface of the entry to the ligand-binding site are indicated. **(B)** Structure of pioglitazone detailing interaction sites with the ligand binding domain of human PPARγ (Structure 2XKW from RCSB, Mueller et al., 2014).

A broad array of PPAR activators were screened, identified and studied in cellular bioassays that measured activity in the native context of the functional PPAR/RXR heterodimer (e.g., Keller et al., 1997; Krey et al., 1997; Devchand et al., 1999). However, some studies also demonstrated the utility of cellular assay systems using homodimeric PPAR-LBD fusion proteins (e.g., Forman et al., 1997; Kliewer et al., 1997). The first report on crystal structures of human PPARγ-LBD was an extremely instructive breakthrough (Nolte et al., 1998; **Figure 4A**).

Consistent with its classification as a nuclear hormone receptor (NHR), the apo-ligand-binding pocket of PPARγ is buried in a core created by layers of alpha helices that form a large T-shaped cavity from the C-terminus to a β-sheet lying between helix 3 and helix 6. Interestingly, compared to other NHRs, PPARγ contains an additional helix H2’ that results in a unique placement of helix 2 and shifting of helices H3, H7 and H10. This arrangement effectively creates easier access for ligands as well as a large hydrophobic scaffold for ligand binding. Key hydrophilic amino acids (D^243^, E^290^, R^288^, and E^295^) line the surface entry point.

Analyses of the X-ray crystal structure of a ternary complex containing rosiglitazone provided the basic frame of how the central benzene ring, thiazolidinedione head group, pyridine ring and carboxylic acid of a glitazone drug are positioned in a U-shaped conformation deep in the pocket of the protein (Nolte et al., 1998). The crystal structure of pioglitazone in human PPARγ-LBD is consistent with these findings in hydrogen bonds to the carbonyl head group of the TZD ring, involvement of amino acids 285–364 in hydrophobic interactions to the central benzene ring and the pivot role of C^285^ for positioning of the pyridine ring (Mueller et al., 2014; **Figure 4B**). The TZD head group of pioglitazone interacts with helix 12 to lock the protein in a conformation that facilitates interaction with nuclear co-factors (Lee et al., 2017). This structural aspect of an “activated” form of the receptor depicts the previously deduced function of the LXXLL motif in PPAR-cofactor interactions that were exploited for ligand discovery (Krey et al., 1997) and for understanding mechanism of action of eicosanoid mimetic compounds (Devchand et al., 1999). A detailed rationale for binding of pioglitazone to PPARγ might facilitate parsing drug selectivity and responsiveness in patients (Agostini et al., 2018).

The crystals of human PPARγ-LBD contain homodimers that are not in themselves considered functional structures *in vivo*, but do provide guidance on PPAR-RXR heterodimer interactions. For example, the PPARγ-LBD homodimer reveal coiled-coil interactions between helix 10 of each monomer, with involvement of the amino acids F^432^, A^433^, L^436^, and T^440^ in forming salt bridges (Nolte et al., 1998).

In summary, the X-ray crystal structures of human PPARγ-LBD depicted many of the structure-function relations that were predicted based on didactic mutagenesis studies and detailed rationale for the promiscuity and selectivity of PPARs for ligands. Furthermore, delineating the similarities and differences of PPARs to other NHRs has been an important step toward understanding the biological roles of PPARs and how to manipulate them with drugs, like pioglitazone.

### Global Reach of Pioglitazones

**FIGURE 5:**
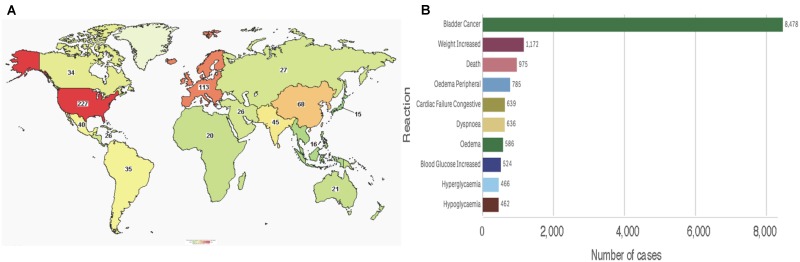
Pioglitazone in patients **(A)** Map showing number of pioglitazone trials that used subjects from each country (source: www.clinicaltrials.org). **(B)** Total reported reactions in the FDA adverse events reporting system for ACTOS from 1999 to December 31, 2018 (source: FAERS).

To date, the clinicaltrials.gov database includes 469 studies using pioglitazone, of which 433 are registered as interventional studies and 36 as observational. Already 154 of these trials have been completed or terminated. Cumulatively, these studies recruited human subjects from all over the world, albeit the majority of 229 trials are located in the United States alone (**Figure 5A**). Treatment categories range widely from “Bacterial and Fungal Diseases” through “Heart and Blood Diseases” to “Wounds and Injuries.” Today, 51 of these studies are active, with main concentration on chronic disease and cancers. Clearly, the pace of growth of pioglitazone use and the breadth of its reach is indicative of a successful medication for global diseases.

Consistent with its approval on the FDA expedited track, the safety of the drug is reflected post-marketing. As of December 31, 2017, the FDA adverse events reporting system (FAERS) indicates that since its approval by the regulatory agency in 1999, ACTOS^@^ logs 18,197 total cases with 14,733 serious cases including deaths. Interestingly, the FAERS Public dashboard shows a peak number of 4,774 (26.24%) reported in 2015. An overwhelming number of total reports indicated bladder cancer, however, various events associated with metabolic and cardiac reactions were also noted (**Figure 5B**). While the scientific basis of cause-and-effect behind these patient reports have been deemed idiosyncratic – for the most part elusive - a rational argument can be made for reflection on the benefits of pioglitazone despite its risks (Soccio et al., 2014).

In summary, pioglitazone has been used globally and is being positioned to increase its reach in patient population. On balance, the information on ongoing clinical trials and the reported adverse effects indicate that the benefits of pioglitazone use outweigh the risks – for now.

### The Long Pioglitazone Road

After decades of didactic pharmacology on clofibrate-based drugs, scientists at Takeda discovered glitazones as a novel class of drugs that improved hyperlipidemia and hyperglycemia by increasing insulin sensitivity in peripheral tissues. The glitazones (troglitazone, rosiglitazone, and pioglitazone) were the first drug to be approved on the FDA expedited track as treatment for the unmet need of T2D patients. These drugs have faced challenges with every advance in the clinic but now, ∼20 years later, pioglitazone is a generic drug and it is being tested for alternate medical uses throughout the world, primarily for prevention and treatment of multifactorial chronic diseases like cancers, diabetes, and metabolic syndromes. We are still far from understanding the mechanisms of action of pioglitazone perhaps in part because the molecular targets appear to be involved in adaptive homeostasis, and therefore dynamic in nature. Pioglitazone’s primary target, identified as PPARγ, reveals a nuanced regulation of transcriptional activity with very tangled networks of complex molecular interactions. Most medications act as inhibitors and dampen over-active responses (e.g., anti-histamines, sulphonylureas, propranolol). Pioglitazone is an agonist of a nuclear receptor, thus has presented different challenges in predicting downstream effects of the drug in patients. Furthermore, the activity of PPAR are not only tissue and context dependent but also dependent on health state and environmental cues. This dynamic complexity of PPAR function offers optimism for treatment of other complex multifactorial diseases, like cancers and late-onstage metabolic diseases. As we gather data and develop a better understanding of both the diseases and the mechanisms of drug action, the success of pioglitazone as a life-saving drug probably lies in selecting the right patient and right time for the right dose of this medicine.

Clearly, in the search for opportunity for increased use of pioglitazone in various disease contexts around the world, the lag in understanding of mechanisms of action of the glitazones must be corrected. The molecular dynamics of adaptive homeostasis creates an attractive and moving target for the use of pioglitazone in safe and effective treatments of spectrums of patients around the globe. It is a long road traveled, and hopefully a long road ahead for pioglitazone to benefit mankind.

## Author Contributions

PRD wrote the manuscript. All authors contributed to its conception and final edits.

## Conflict of Interest Statement

The authors declare that the research was conducted in the absence of any commercial or financial relationships that could be construed as a potential conflict of interest.

## Footnotes

1 www.who.org

2 http://www.niddk.nih.gov

3 clinicaltrials.gov
